# Differences in Obese and Underweight 5- to 8-Year-Old Children’s Physical Fitness and Motor Skills and Associations With Body Composition: The ExAMIN Youth and BC-IT SA Study

**DOI:** 10.1177/00315125251348493

**Published:** 2025-06-10

**Authors:** Carli Gericke, Anita Elizabeth Pienaar, Barry Gerber, Makama Andries Monyeki

**Affiliations:** Physical Activity, Sport and Recreation (PhASRec), Faculty of Health Science, Potchefstroom Campus, 56405 North-West University, Potchefstroom, South Africa

**Keywords:** health-related physical fitness, motor-related physical fitness, motor skill, overweight, underweight

## Abstract

**Background:** Malnutrition, encompassing underweight (UW) and overweight or obesity (OW/OB), is a global health challenge that significantly impacts children’s physical fitness and motor development. **Purpose:** As limited research exists on these relationships in young children, this study investigated differences in health-related physical fitness (HRPF), motor-related physical fitness (MRPF) and motor skills (MS) in UW and OW/OB children and associations between these fitness characteristics and body composition parameters. **Research Design:** A cross-sectional study design was used. **Sample:** 298 children (150 boys, 148 girls; mean age 6.84 ± 0.96 years) was drawn from the ExAMIN Youth SA and the BC-IT studies in the North-West Province of South Africa. **Data Collection and Analysis:** Measurements included anthropometric measures, body composition assessed using bioelectrical impedance analysis, and evaluations of HRPF, MRPF, and MS). Data were analysed using SPSS (v. 26.0). **Results:** In the group, 26% were underweight, 11.1% overweight, and 8% obese. OW/OB children had poorer physical and motor fitness (*p* < .05) and motor skills (*p* > .05) than normal-weight (NW) peers, while underweight children significantly outperformed both OW and OB and normal-weight children. All body composition parameters were largely and negatively associated with strength, aerobic capacity (r > 0.5), speed, agility and balance in obese children. Fat-free mass, body mass index (BMI), and waist circumference revealed positive, more minor and inconsistent associations (r > 0.2) in NW and UW children. Waist circumference (WC) and FFM correlated positively with balance and catching in UW and NW children, with negative correlations between balance and BMI and WC in overweight and obese groups. **Conclusions:** These different degrees of associations with physical and motor fitness and motor skills in under- and overweight children are important when designing early interventions to prevent childhood obesity.

## Introduction

Malnutrition in all its forms remains one of the most significant global health challenges, affecting both children and adults (WHO, 2021). Malnutrition encompasses undernutrition—characterised by wasting, stunting, and underweight (UW)—and overnutrition, which includes overweight (OW) and obesity (OB). Both forms of malnutrition have significant developmental and health consequences in childhood and adolescence (Kamruzzaman et al., 2021; Modjadji & Madiba, 2022; Monyeki et al., 2015), which can persist into adulthood (Deren et al., 2018). In young children, malnutrition is linked to developmental delays (Kahn et al., 2021), cognitive impairments (Pelletier & Frongillo, 2003), poor academic performance (Pelletier & Frongillo, 2003) and also contributes to an increased risk of mortality (Wolnicka et al., 2016).

Underweight is defined as thinness resulting from rapid weight loss or an inability to increase body mass (Cole et al., 2007). Stunting, another form of undernutrition, is defined as height-for-age more than two standard deviations below the WHO Child Growth Standards median, signifying the results from prolonged inadequate nutrition (de Onis & Branca, 2016), and is considered to be the long-term effect of nutritional problems in a community (WHO, 2021). OW/OB is another form of malnutrition defined by excessive fat accumulation that poses health risks (WHO, 2021). Children’s physical growth is regulated by two primary factors: genetics and the environment (Saleemi et al., 2001). The literature points to child growth as an interplay between economic, demographic, environmental, and cultural changes in a society in predicting and determining the nutritional status of a population (Bourne et al., 2001; Monteiro et al., 2002).

Worldwide statistics indicate that 5.6% of girls and 7.8% of boys are classified as obese based on BMI, as cited in a report by the Obesity Evidence Hub (Georgousakis, 2019). According to WHO (2023), among children under five years old, 41 million are OW/OB, 155 million are stunted, 52 million are wasted, and 17 million are severely wasted. It is well-documented that the prevalence of OW/OB is typically higher in developed countries. However, there has been a recent rapid transition from UW to OW/OB in low- and middle-income countries (LMICs), such as South Africa (Abarca-Gómez et al., 2017) of South African children under five were underweight, while 13% were classified as OW (Statistics South Africa, 2022). Overnutrition, or OB, is also more prevalent in young South African boys than in girls (15% vs. 11%) (Children Count, 2019). It is (Allen & Kelly, 2019) evident that malnutrition, in all its manifestations, imposes several risks to diet-related non-communicable diseases in children. It is estimated that eliminating malnutrition can reduce the global disease burden by 32% (WHO, 2023). Nutritional transition in South Africa is complex and is characterised by urban migration and economic and social transitions, all contributing to rising OW/OB rates (Kahn et al., 2021). Addressing these trends is critical to mitigating developmental risks, including physical, motor, and academic difficulties and health-related problems in early childhood (Allen & Kelly, 2019; Meriem et al., 2020; Wilhelmsen et al., 2023).

Motor-related physical fitness (MRPF), health-related physical fitness (HRPF), and motor skills (MS) are essential markers of general health and development in children and adolescents, all of which can be influenced by malnutrition (Raghuveer et al., 2020; Robinson et al., 2015; Ruiz et al., 2009). Since these are key components of an active and healthy lifestyle, poor MS and low PF in children are major threats to future public health, as clear declines have been reported in children and adolescents over the last several decades (Drenowatz et al., 2021; Tomkinson et al., 2019). For example, 74% of Czech children indicate ‘below normal’ muscular fitness (Müllerova et al., 2015), while less than half of US adolescents achieve healthy fitness levels (Gahche et al., 2014). Concurrently, the prevalence of OW/OB in children is increasing, with a significant effect on the current and future health of children (Llewellyn et al., 2015) due to negative influences thereof on MS and PF. Utesch et al. (2018) reported a strong positive relationship between MS and PF, which strengthens with age, suggesting that these components influence OB risk (Stodden et al., 2008; Utesch et al., 2018). Furthermore, OW/OB is associated with poor gross motor development, endurance, PA, PF and MS, and musculoskeletal constraints in children (Baard, 2014; Choukem et al., 2020; de Waal & Pienaar, 2021). Studies consistently report low motor competence in OW/OB children (D’Hondt et al., 2012; Cheng et al., 2016; Lima et al., 2019, 2021), with potential contributing factors including urban lifestyles, increased screen time, and the shift from active transport, particularly in rural areas (Choukem et al., 2020; Prista et al., 2016).

UW has also been shown to negatively influence some measures of PF (Huang & Malina, 2007). Poor nutritional status is negatively associated with physical activity, contributing to lower PF (Bakaloudi et al., 2020; Monyeki et al., 2005). Studies have shown that UW boys and girls aged six to 11 years have a higher risk of having a lower PF index than their normal-weight peers (Fiori et al., 2020). A study on 14-year-old South African children reported a strong, significant positive association between PF and BMI in UW girls, with a non-significant, weaker positive relationship in UW boys (Monyeki et al., 2012).

This background underscores the association between UW and OW/OB and decreased physical capacity, which in turn is linked to reduced cardiorespiratory fitness (CRF), muscle strength, and speed of movement (Shang et al., 2010). Addressing malnutrition is, therefore, critical to preventing developmental limitations and poor health during early childhood.

However, there is still a lack of research on the relationship between malnutrition and fitness-related characteristics in children, and mainly how the fitness and motor skills of boys and girls at a young age are influenced by these conditions in the South African context. Studies investigating these using bioelectrical impedance analysis (BIA) also remain limited. Such studies are needed as BIA provides information on additional variables such as FFM, FM in kg and FM%, while BMI is limited in providing such information.

Against this background, this study aimed to determine differences in the HRPF, MRPF, and MS of five-to-eight-year-old obese and underweight children. Secondly, to investigate relationships of body composition with HRPF, MRPF, and MS parameters in UW and OW/OB children residing in the North-West Province of South Africa, including associations with BMI, WC, FM%, and FFM.

## Methodology

### Research Design

This cross-sectional study uses available data from the ExAMIN Youth and the Body Composition (BC) by Isotope Techniques (BC–IT) studies. The ExAMIN Youth Study is an analytical, multidisciplinary, observational cohort study designed to investigate the interplay between body composition, nutrition, physical activity, as well as biomarkers related to psychosocial stress and cardiovascular function and salivary biomarkers in ∼1100 children (aged five- to eight-years) attending 10 public primary schools within the Kenneth Kaunda district (Potchefstroom, Klerksdorp) in the North-West Province, South Africa (Kruger et al., 2020; Kruger et al., 2021), The BC–IT study examined the relationships between more complex markers (using a stable isotope method and Bioelectrical Impedance Analysis (BIA) and more indirect measures of body composition (using anthropometric variables), and objective and subjective measures of physical activity, physical fitness and motor skills and their relationships with other health-related determinants, among five to eight-year-old South African children.

The methodology of this sub-study is related to the methodology that describes the original study (Kruger et al., 2020; Moeng-Mahlangu et al., 2020).

### Research Group

This subsample included 298 children (150 boys and 1438 girls) aged between five and eight years with complete data on BC using an isotope technique [the Body Composition–Isotope Technique (BC–IT) study; 2018/2019]. All children in the subsample had to have complete data on the Deuterium Dilution Method (DDM), and motor and health-related fitness and motor skills measurements to be included in the study. To study these variables, five primary schools were randomly selected from 26 schools in the JB Marks municipal area (Potchefstroom) in the Kenneth Kaunda district of the North-West Province, South Africa. These schools represented different school quintiles (3 (low SES) – 4 and 5 (high SES). The Generalised Linear Model for Analysis of Variance was used to calculate the statistical power of the appropriate sample size for a power of 0.80 and a level of 0.05 at a CI of 95%. Every third child on each class list was selected and invited to participate in the study. Only those with parental consent and child assent were allowed to participate. The mean age of this subsample was 6.84 (±0.96) years, including participants in the following age groups: five years (*n* = 26), six years (*n* = 86), seven years (*n* = 95), and eight years (*n* = 91).

### Ethical Approval

The research was performed following the Declaration of Helsinki guidelines. The Health Research Ethics Committee (HREC) of the Faculty of Health Science at the North-West University, Potchefstroom, SA, granted permission for the observational cohort/follow-up study, the ExAMIN Youth SA (NWU-00091-16-A1), the cross-sectional BC-IT study (NWU-00025-17-S1), and this sub-study (NWU-00457-20-A1). Approval was also given by the Department of Basic Education, school principals, parents and children. Written parental or legal guardian consent had to be obtained, while the participants had to provide verbal or written assent based on age to participate in the study. The parental/ legal guardian consent form explained what would be expected of the participants and outlined the risks involved. On the day of data collection, the steps and procedures followed during the data collection process were explained briefly to the participants.

### Measuring Instruments

#### Anthropometric Measurements

The International Society for the Advancement of Kinanthropometry (ISAK) protocol was used to determine the anthropometric measures of height (cm) and weight (kg) of the participants (Stewart et al., 2010). Certified level 2 kinanthropometrists took these measurements. The measurements were taken in separate rooms for the boys and girls to ensure privacy. A Seca 213 stadiometer (Holstein Limited, Crosswell, Crymych, UK) measured height to the nearest 0.1 cm. Participants were barefoot and standing upright with their heads in the Frankfort plane, after they had inhaled. Weight was measured to the nearest 0.1 kg with a Seca 813 digital scale (Beurer Ps07 Electronic Scale, Ulm, Germany), while participants wore minimal clothing with no shoes. Waist circumference was measured between the narrowest point of the abdomen and the lower costal (10th rib) with a Lufkin metal tape (Cooper Industries, U.S.A.) to the nearest 0.1 cm. BMI was calculated as weight divided by height squared (weight in kg/height squared in meters), and BMI z-scores were calculated relative to WHO reference data. The WHO BMI z-score categories that were used were UW: −2 SD from the median; normal weight: −2 SD to +1 SD; OW: more than +1 to +2 SD; and OB: more than +2 SD (de Onis, 2007).

### Body Composition by Bioelectrical Impedance Analysis

Body composition (BC) was assessed using BIA with a Bodystat 1500 MDD following the protocol provided by the manufacturer. Children were asked to refrain from exercise or taking a sauna/playing in the sun within 8 hours of the procedure. Frequency of measure was set at 50 kHz, with height, sex, age and body mass entered manually. Body mass was automatically adjusted using 0.5 kg for clothing mass in all participants. Participants were asked to remove jewellery and belts containing metal or metal-rimmed glasses. Participants were asked to lie down quietly and without motion on a non-metal examination table or bed on their back with arms flexed to the side and thighs not touching. Detection electrodes were placed at the pisiform prominence of the wrist and the anterior surface of the true ankle joint after wiping with moist antiseptic towel (Right side as this is recommended) (Schoeller et al., 1980).

The Bodystat software uses inbuilt prediction equations to produce an output specifying total body water (TBW) in litres, FFM (kilograms), BF mass (kilograms) and BF%, as well as impedance, resistance and reactance readings. The existing BIA equations developed by Luke et al. (1997) were used to estimate total body water (TBW). Fat free mass (FFM) was calculated using age- and gender-specific Lohman hydration factors for children (International Atomic Energy Agency (IAEA), 2010; Wang et al., 1999). Children were classified as normal (FM% 14.9% to 24.9), UW/thinness FM%<14.5%, OW (FM% > 30 & 34.9) and OB (FM% >35%) in line with the McCarthy et al. (2006) and Williams et al.’s (1992) cut points as was applied in the recent publication from the BC–IT study by Moeng-Mahlangu et al. (2020).

### Physical and Motor Fitness Tests

This testing protocol included tests from different test batteries to assess HRPF, MRPF and MS (Livonen & Sääkslahti, 2013; Plowman & Meredith, 2021; Pienaar et al., 2016). Three health-related physical fitness (HRPF) characteristics were tested, including the 20-m (m) shuttle run test (20-m SRT), predicted $\dot{V}$ O_2max_ in millilitres of oxygen used in 1 minute per kilogram of body weight (ml/kg/min), and leg strength. The 20-m SRT is a valid and recognised endurance test that shows reliability in children aged six to 16 (r = 0.89). The test involves running back and forth across a 20-m distance (Plowman & Meredith, 2013). The starting speed is 8.0 km/h, with a 0.5 km/h rise every minute, paced by beeps on a stereo. A final score is taken when a participant drops out because of exhaustion or cannot cross the 20-m line at the point of the beep for two consecutive 20-m lengths. An indirect aerobic capacity score ( $\dot{V}$ O_2max_) was calculated using the FitnessGram equation, including field test scores, age, sex, and BMI (Plowman & Meredith, 2013). The following equation was used to convert the attained beep levels to predict aerobic capacity: $\dot{V}$ O_2max_ 45.619 + (0.353*Pacer laps) – (1.121*age).

Motor-related fitness was tested using running speed and agility measurements. A 10-m and 20-m speed test determined running speed. Electronic timing gates (Smartspeed, Fusion Sports, Summer Park, Australia), which have a reliability of 0.9 in children aged six to 11 years, were used. After an acoustic signal, the participant starts the 20-m run from a standing position. The time in seconds to complete the 10-m and 20-m sprint tests was recorded as quantitative measures of running speed, where the best of two trials was scored. Agility was also assessed quantitatively using a two-legged jumping sideward test from the Körperkoordinationstest für Kinder test battery (KTK) (Livonen & Sääkslahti, 2013), where the number of successful sideways jumps was scored in 15 seconds.

#### Test of Gross Motor Development-2 (TGMD-2)

Process (quality) and product (quantity) performance of motor skills were evaluated through running, jumping, catching, kicking, and balancing. These skills were selected to obtain a comprehensive overview of the motor skills abilities of the participants. Four tests that represent two locomotor skills (running and jumping) and two object control skills (catching and kicking) were used from the Test of Gross Motor Development (TGMD-2) protocol (Ulrich, 2000) to assess motor skills qualitatively (process). Jumping represented a qualitative measure of leg strength in HRPF, and running was a qualitative measure of running speed in MRPF. The catching and kicking skills represented qualitative measures of object control motor skills. These four skills are also the most common ones often chosen by researchers and are most relevant to typical South African sports and game activities. It is not uncommon for researchers to select only a few of these skills for their studies. Following the TGMD-2 protocol, the skills of running, jumping, catching and kicking were demonstrated. Then, two attempts were allowed and scored according to specific sub-criteria (0 = no mastery, 1 = mastery), after which the scores for each skill were added together. The following sub-criteria of the TGMD-2 protocol were used for these process (quality) assessments (running = four sub-criteria; jumping = four sub-criteria; catching = three sub-criteria and kicking = four sub-criteria), as described elsewhere (Ulrich, 2000). Balancing was scored qualitatively out of 3 (1 = initial phase, 2 = elementary phase, 3 = mature phase) using the Kinderkinetics protocol described elsewhere (Pienaar et al., 2016). A product assessment evaluates a movement’s outcome, typically identified as a quantitative score (e.g., speed, distance, or number of successful attempts). Product-oriented evaluations of this study protocol included running speed in seconds by scoring the best of two trials to complete a 10-m and 20-m run test. Catching and kicking accuracy were scored out of five attempts using the TGMD-2 protocol (distances between the tester and the participant in the catching skills and the distance to the kicking target of 1.5 cm wide). The distance jumped in the horizontal jumping test (SBJ) of the TGMD-2 was used as a quantitative measure for jumping. Two trials were allowed, and the best trial was recorded in centimetres. This test was performed on a non-slippery mat explicitly designed for horizontal jumping.

#### The Körperkoordinationstest für Kinder Test Battery (KTK)

Two of the four KTK test items (Livonen & Sääkslahti, 2013; Rudd et al., 2016) were used to obtain quantitative scores of balance and agility. Balancing was tested by walking backwards along a balance beam with decreasing width, from 6.0 cm to 4.5 cm–3.0 cm, counting the number of successful steps. Agility (MRPF) was tested by the number of two-legged jumps performed successfully sideward in 15 seconds.

Senior researchers and honours students in Kinderkinetics tested all the participants. Researchers in the data collection team were all trained beforehand. The same evaluators were used for each test item to reduce tester variability and improve the reliability of the results. The participants completed all tests by rotating between stations, with different stations manned by honours students and managed by senior researchers. The 20-m shuttle run was done after all measures were taken to prevent exhaustion influences on the various tests. As this is a challenging test for young children to complete, all efforts possible were made to obtain a valid testing outcome, including the whole research team in this testing. Some ran with the participants to assist them with pacing, while others encouraged them from the side for their continued effort. On the day of testing, all the participants were transported by bus from their school to the PhASRec laboratory at the North-West University, tested, and then returned to the school before the end of the school day.

### Statistical Analysis

The data were analysed using the Statistical Package for Social Sciences (SPSS v 26.0). The normality of the data was determined using the Shapiro-Wilk test, which was acceptable in body composition parameters. Descriptive statistics were used to characterise the research sample, including frequency percentages, means, and standard deviations. Due to the non-normality in HRPF and MRPF data, Spearman correlation coefficients (Rho) were calculated to examine relationships between UW and OW and HRPF, MRPF, and MS. A *t* test was used to determine differences between boys and girls and an ANOVA with a post-hoc Bonferroni adjustment was used to determine the influence of UW, OW and OB on HRPF, MRPF, and MS in five- to eight-year-old children, dividing the group into underweight, normal weight, overweight and obese groups. For the interpretation of correlation coefficients, the Cohen (1988) cut points were used with the following interpretations: r = 0.1–0.29 is considered a weak correlation; r = 0.3–0.49 a moderate correlation; and r = 0.5–1.0 a strong correlation.

## Results

In the group of 298 participants (150 boys and 148 girls) with a mean age of 6.84 years (±0.96), body fatness categories based on the BIA showed 26% (*n* = 79) of the group as UW, 11.1% (*n* = 33) OW and 8.0% (*n* = 24) OB (Table 1).

**Table 1.** Body Fatness Categories in the Group and Age Distribution of the Participants.

Body fatness categories (BIA)	*N*	%
Normal body fat	162	54.4
Underweight/Thinness	79	26.5
Overweight	33	11.1
Obese	24	8.0
Total	298	100

*N* = Total number of participants, % = percentage of participants.

Table 2 compares body composition parameters, health-related physical fitness, and motor-related physical fitness according to these different body fatness categories (overweight, obese, normal weight and underweight). These results show that the OB group was significantly taller, heavier, and had a higher body fat percentage than all other groups. Both the OW and OB groups had higher adipose tissue, body fat percentage (BF%), and fat mass (FM) compared to UW and NW children (*p* < .05).

**Table 2.** ANOVA Analysis for Body Composition, HRPF, HRPF and Motor Skills Parameters According to Body Fatness Categories.

Body Composition Parameters	Total	Underweight^a^	Normal^b^	Overweight^c^	Obese^d^
	Mean ± SD	Mean ± SD	Mean ± SD	Mean ± SD	Mean ± SD
Body height (cm)	122.61 ± 7.98	122.51 ± 7.71*^d^	122.05 ± 7.86*^d^	121.72 ± 9.11*^d^	127.99 ± 6.23*^a,b,c^
Body weight (kg)	24.50 ± 6.44	22.86 ± 4.01*^d^	23.45 ± 5.00*^d^	25.83 ± 7.02*^d^	35.15 ± 9.96*^a,b,c^
BMI (kg/m^2^)	16.10 ± 2.61	15.13 ± 1.32*^c,d^	15.60 ± 1.62*^c,d^	17.13 ± 2.48*^a,b,d^	21.22 ± 4.63*^a,b,c^
WC (cm)	54.93 ± 6.93	53.55 ± 4.48*^d^	53.44 ± 5.22*^c,d^	56.75 ± 6.93*^a,d^	66.99 ± 10.74*^a,b,c^
Body fat (BF) (%)	23.53 ± 8.11	13.84 ± 4.09*^b,c,d^	23.99 ± 2.83*^a,c,d^	32.25 ± 1.24*^a,c,d^	40.24 ± 3.29*^a,b,c^
Fat (kg)	6.07 ± 3.57	3.46 ± 1.94*^b,c,d^	5.64 ± 1.47*^a,c,d^	8.41 ± 2.32*^a,b,d^	14.34 ± 5.03*^a,b,c^
Lean mass (kg)	18.52 ± 3.99	19.70 ± 3.40*^b^	17.81 ± 3.73*^a^	17.59 ± 4.73*^d^	20.72 ± 4.86*^b,c^
TBW (kg)	14.75 ± 5.35	15.83 ± 6.13	14.02 ± 4.49	14.83 ± 7.45	16.08 ± 3.72
HRPF					
Standing broad jump (cm)	113.11 ± 18.55	123.71 ± 18.48*^b,c,d^	111.71 ± 17.59*^a,c^	102.88 ± 9.96*^a,b^	101.74 ± 17.23*^a^
PACER laps	27.51 ± 12.96	31.72 ± 13.92*^c,d^	27.86 ± 12.48*^c^	19.39 ± 9.48*^a,b^	22.46 ± 10.66*^a^
$\dot{V}$ O_2max_ (mL/kg/min)	47.19 ± 4.30	48.62 ± 4.56*^c,d^	47.31 ± 1.06*^c^	44.30 ± 3.18*^a,b^	44.73 ± 3.65*^a^
MRPF					
10 m speed (sec)	2.41 ± 0.26	2.29 ± 0.17*^b,c,d^	2.42 ± 0.27*^a,c^	2.58 ± 0.28*^a,b^	2.47 ± 0.17
20 m speed (sec)	4.34 ± 0.45	4.14 ± 0.30*^b,c,d^	4.35 ± 0.43*^a,c^	4.63 ± 0.65*^a,b^	4.50 ± 0.36*^a^
Running qual (total)	7.18 ± 1.12	7.36 ± 0.89*^c^	7.22 ± 1.08*^c^	6.60 ± 1.41*^a,b^	7.04 ± 1.40
Agility (total)	42.25 ± 13.67	44.80 ± 12.25*^c^	42.47 ± 14.02	36.00 ± 14.71	41.04 ± 12.25
Motor skills					
Balance quant (total)	35.51 ± 11.52	36.60 ± 10.77	36.27 ± 11.48	32.84 ± 13.59	30.50 ± 9.78
Balance qual (total)	2.34 ± 0.58	2.43 ± 0.54	2.32 ± 0.57	2.24 ± 0.70	2.31 ± 0.60
Catching quant (total)	3.41 ± 1.54	3.53 ± 1.45	3.32 ± 1.63	3.03 ± 1.53*^d^	4.12 ± 0.99*^c^
Catching quant (total)	5.21 ± 0.95	5.33 ± 0.81	5.10 ± 1.06*^c^	5.12 ± 0.82	5.67 ± 0.48*^b^
Kicking quant (total)	2.95 ± 1.33	2.92 ± 1.24	3.04 ± 1.38	2.79 ± 1.34	2.58 ± 1.17
Kicking qual (total)	7.19 ± 1.25	7.51 ± 0.98	7.22 ± 1.19	6.81 ± 1.61	6.54 ± 1.59

*N* = sample size; cm = centimetre; kg = kilogram; SD = standard deviation; FM% = fat-mass percentage; BMI = body mass index; WC = waist circumference; TBW = total body water; sec = seconds; % = percentage; $\dot{V}$ O_2max_ = maximal oxygen consumption; mL/kg/min = millilitres of oxygen used in 1 minute per kilogram of body weight; * = statistical significance differences for group comparison (*p* < .05); HRPF = health related physical fitness; MRPF = motor related physical fitness. Letter after means show significance between groups.

Obese, including overweight children, were also the groups with the poorest scores (*p* < .01) in all HRPF (standing broad jump, PACER and $\dot{V}$ O_2_max), MRPF (speed and agility) and almost all the motor skills, except for catching, where obese children received superior qualitative and quantitative scores. The OB group exhibited the lowest performance in SBJ *p* < .05) and the poorest outcomes in both PACER laps and predicted $\dot{V}$ O_2_max. The UW group had the lowest BMI (15.13 ± 1.32) of the groups, while the NW group exhibited significantly lower lean mass (17.81 ± 3.73 vs. 19.70 ± 3.40) and total body water (TBW) (14.02 ± 4.49 vs. 15.83 ± 6.13), *p* > .05 compared to the UW group. The health-related physical fitness of the UW group showed superior lower-body strength, with a significantly greater standing broad jump (SBJ) distance (123.71 ± 18.48 cm) compared to the NW (111.71 ± 17.59 cm), OW (102.88 ± 9.96 cm), and OB (101.74 ± 17.23 cm) groups (*p* < .001). Additionally, the UW group significantly outperformed the OW and OB groups in aerobic capacity (PACER laps) and predicted $\dot{V}$ O_2_max (*p* < .001). UW children also had insignificant higher aerobic and VO_2_ max values than the NW group (*p* > .05). Performance in speed (10 m, 20 m sprint), running quality, and agility, all tests of motor-related physical fitness, again showed significantly higher scores in the UW group (*p* < .001) compared to the OW and OB groups. Additionally, the UW group outperformed the NW group in both qualitative and quantitative balance and kicking assessments. The NW group performed better than the OW and OB groups but remained inferior to the UW group across all HRPF measures. They also displayed significantly lower 10- and 20-m speed and agility scores than UW children and insignificant balancing, catching, and kicking motor skills scores (*p* > .05) than the UW group. Interestingly, no significant differences in TBW were observed among the groups (*p* > .05).

Table 3 displays the results of a correlation analysis between BMI, WC, BF%, and FFM on HRPF, MRPF and MS parameters in the different weight status groups. In the obese group, the HRPF parameters (SBJ, PACER laps, and predicted $\dot{V}$ O2max), BMI, WC, and FFM all showed significant inverse, moderate-to-large negative correlations. FM% had the highest association with strength (r = −0.62), while BMI correlated the highest with aerobic capacity [PACER and VO_2_ max (r = −0.73 and r = −0.71)]. In UW children, only strength correlated with most body composition parameters in the UW group, except BF%, at a moderate level (r = 0.34- r = 0.52). Here, FFM correlated the highest and positively with strength (r = 0.51). In UW children, aerobic fitness (PACER and VO_2_ max) correlated only with FFM (r = 0.27 and 0.22). Speed and agility also correlated higher with all body composition in the obese group than in UW children, while UW children did not show associations with BF%.

**Table 3.** Correlations (r) Between BC, Fitness and Motor Skills Parameters in UW and OW/OB.

	Normal (*n* = 162)				Underweight (*n* = 79)				Overweight (*n* = 33)				Obese (*n* = 24)			
Variables	BMI	WC	BF%	FFM	BMI	WC	BF%	FFM	BMI	WC	BF%	FFM	BMI	WC	BF%	FFM
HRPF																
SBJ (cm)	0.16^a^	0.33^b^	−0.10	0.39^b^	0.34^b^	0.48^b^	−0.20	0.51^b^	−0.05	0.02	0.01	0.10	−0.56^a^	−0.46^b^	−0.62^b^	−0.42^a^
Pacer laps	−0.04	0.16^a^	−0.06	0.19^a^	0.03	0.12	−0.06	0.27^a^	0.31	0.30	0.04	0.31	−0.73^b^	−0.65^b^	−0.42^a^	−0.66^b^
$\dot{V}$ O_2_max	−0.15^b^	0.02	−0.11	0.02	0.05	0.12	−0.07	0.22^a^	0.12	0.09	0.08	0.10	−0.71^b^	−0.59^b^	−0.35^a^	−0.65^b^
MRPF																
Speed 10 m (sec)	−0.16^a^	−0.39^b^	0.11	−0.37^b^	−0.28^a^	−0.39^b^	0.04	−0.34^a^	−0.37^a^	−0.45^b^	0.13	−0.54^b^	0.53^b^	0.47^a^	0.22	0.52^b^
Speed 20 m (sec)	−0.14	−0.37^b^	0.12	−0.41^a^	−0.27^a^	−0.38^b^	−0.02	−0.43^b^	−0.45^b^	−0.54^b^	−0.15	−0.61^a^	0.59^b^	0.53^a^	0.33^b^	0.55^a^
Running quality (tot)	0.06	0.13	0.05	0.02	0.08	0.10	−0.13	0.18	0.15	0.12	0.24	0.05	−0.42^a^	−0.51^a^	0.03	−0.46^a^
Agility (total)	0.14	0.25^b^	−0.003	0.23^b^	0.27^a^	0.35^b^	0.19	0.26^a^	0.59^b^	0.53^b^	0.33^b^	0.55^a^	−0.28	−0.38^a^	0.18	−0.48^a^
Motor skills																
Balance quantity	0.10	0.17^a^	−0.07	0.25^b^	−0.14	−0.17	0.09	−0.7	0.05	0.04	−0.33^b^	−0.04	−0.50^a^	−0.51^a^	−0.37	−0.36^a^
Balance quality	0.24^b^	0.33^b^	−0.07	0.33^b^	0.21	0.29^b^	0.18	0.24^a^	0.08	0.23	0.07	0.06	0.07	0.16	−0.05	0.15
Catch quantity	0.24^b^	0.33^b^	0.06	0.45^a^	0.20	0.26^a^	−0.03	0.42^b^	0.30^a^	0.36^a^	−0.05	0.41^a^	0.19	0.06	0.15	0.17
Catch quality	0.27^b^	0.41^b^	0.05	0.47^b^	0.15	0.28^a^	0.02	0.36^a^	0.47^b^	0.44^a^	−0.32^b^	0.47^b^	0.25	0.23	0.08	0.07
Kicking quantity	0.08	0.05	0.02	0.12	0.04	0.09	−0.13	0.07	0.48^b^	0.33^b^	−0.13	0.22	−0.20	−0.14	−0.18	−0.10
Kicking quality	−0.001	0.08	−0.06	0.02	0.19	0.22	−0.08	0.20	0.03	0.05	0.35^a^	0.07	0.21	0.37^a^	0.07	0.28^a^

^a^Correlation is significant at the 0.05 level (2-tailed).

^b^Correlation is significant at the 0.01 level (2-tailed).

BMI = body mass index; WC = waist circumference; FM% = fat mass percentage; FFM = fat free mass; HRPF = health-related physical fitness; SBJ = standing broad jump; All qualitative measurements = total.

Speed and agility also correlated with all body composition measures in obese children, although at a moderate level. The UW group showed similar associations with body composition, although lower associations were found compared to the obese group. The quality of running of obese children also correlated inversely at a moderate level with BMI (r = 0.-42), WC (r = −0.55) and FFM (r = −0.46), while no such correlations were evident in the UW group. The overweight group showed similar correlations to the obese group, while the NW and UW groups revealed similar correlations in HRPF and MRPF.

Of the motor skills, only the ability to balance while walking on a beam was negatively associated in the obese group with all four body composition parameters. BMI (r = −0.50) and WC (d = −0.51) show the largest associations. Interestingly, kicking quality correlated positively with WC (r = 0.37) and FFM (r = 0.28) in obese children. In the UW group, catching (quality and quantity) and the quality of balancing showed positive correlations with WC and FFM, while no correlations emerged in kicking and the ability to balance (quantitative). Again, mostly similar associations emerged in the NW group compared to the UW group and between the OW and obese groups.

## Discussion

This study examined differences in UW and OB children’s health-related physical fitness (HRPF), motor-related physical fitness (MRPF), and motor skills (MS) and the associations of body composition with these fitness and motor skills characteristics in these children with a mean age of 6.84 years. In this group, 26.5% were underweight, 11.1% overweight, and 8.0% obese. These prevalences align well with national and regional obesity statistics in South African studies. Hall et al. (2019) reported that approximately 13% of under-five-year-old South African children are OW, while regional studies on older children (Moeng-Mahlangu et al., 2020; Otitoola et al., 2020) report similar statistics. Prevalence ranged between 8.8 % overweight and 2.4 % obese (Moeng-Mahlangu et al., 2020), 1.9% underweight, 14.8 % overweight and 2.8% obese (Otitoola et al., 2020). Underweight is reported to be 5.9% nationally, although it is based on BMI (National Department of Health, 2018), while our prevalence is based on BIA (de-Mateo-Silleras et al., 2019). These prevalences confirm malnutrition challenges in many of these young children that again can influence other aspects of their development, including fitness and motor skills development.

Our study confirmed significantly poorer MRPF, HRPF, and MS performance in OW and OB children compared to UW and NW peers. The findings also confirmed that almost all body composition characteristics of OW and OB children were negatively associated with their health—and motor-related fitness and, to a lesser extent, their motor skills. However, the magnitude of the associations differed between fitness and motor skills. Some body composition parameters also revealed higher associations with specific fitness parameters than others. These associations were mostly of large practical significance, especially in HRPF, which represents strength and aerobic capacity capabilities.

Associations of the different body composition parameters with similar fitness and motor skills in UW children were, however, rather positive, showing moderate significance while also revealing associations with most of the motor skills. In HRPF of UW children, only strength was associated with BMI, WC and especially FFM (r = 0.66), while aerobic capacity only showed associations with FFM (r = 0.22 and r = 0.27). Overall, the results revealed moderate to large significant associations between BMI, WC, FFM and MRPF, HRPF, and MS in children with different weight statuses, while BF% were not significantly associated with any of these fitness measures.

The positive associations between standing broad jump and WC and FFM may be attributed to the difference in muscular content in different normal, underweight versus overweight and obese groups (Chen et al., 2022). Although the physiological mechanisms are not yet clarified, it may also be behind the above-mentioned associations of BMI with running and jumping performances. The negative associations observed in the overweight and obese groups could be explained by the differences in the excess of BF% and Fat in kg because this mass is an extra load to be moved while performing the tests (Artero et al., 2010). These associations are supported by the higher correlations of BMI, WC, BF, and FM in the sample, ranging from r = 0.65 to 0.85. It should also be noted that BMI is based on height and needs to be observed when interpreting the associations.

UW children performed significantly better in all HRPF (Standing broad jump, PACER laps, and predicted $\dot{V}$ O_2max_) and MRPF (10 m and 20 m running and agility and running quality) and MS (balance and kicking) than OW and OB children, except for catching. In addition, except for kicking skills, the UW group also performed significantly better in the MRPF, HRPF, and MS tests than the normal weight group. The results also confirmed that higher BMI, WC and FFM negatively affected the running quality of obese children. Higher fat-free mass means more body weight, making running more physically demanding. Heavier children may experience greater impact forces with each stride, which can increase joint stress and make sustained running more difficult, which affects optimal running economy. Nobre et al. (2022) report poor quality in locomotor skills of obese 3-5-year-old children and suggest that being overweight/obese may hinder displacements since antigravity activities are more difficult due to the morphological restrictions to movement within high biomechanical restrictions, which make it more challenging to perform tasks involving changes in the centre of mass. These can decrease running speed and the mechanics of running and, subsequently, the quality of running, which was evident in our obese group.

Overall, the results revealed moderate to large significant associations between BMI, WC, FFM, MRPF, HRPF, and MS, while BF% was not significantly associated with these fitness measures. Body fat mass percentage measures the proportion of body weight that comes from fat, but does not account for muscle mass or overall body weight. Therefore, body fat mass percentage alone does not determine fitness, as multiple factors, such as muscle mass, physical activity, growth stage, and type of testing protocol, can influence this relationship.

Various studies have reported obesity to be associated with poor performance in MRPF, HRPF, and MS levels in children (Cheng et al., 2016; Förster et al., 2023; Ratajczak & Petriczko, 2020; Wyszyńska et al., 2020). Low and very low BMIs were also associated with higher performance in other studies (Haywood et al., 2021; Vandoni et al., 2021; Verbecque-Alva et al., 2022; Xu et al., 2020), which corresponds with our findings affirming better performance in the NW and UW groups compared to children with high BMI levels.

In this study, BMI, WC, and FFM were all largely associated with all HRPF measures except for BF% in obese children. The association of BMI with HRPF (strength and aerobic capacity) in obese children ranged from r = -0.56 to r = −0.73; for WC, it ranged between r = −0.46 (strength) to r = −0.64 (aerobic capacity), FFM between r = −0.35 and r = −0.62 and BF%, between −0.35 and −0.63. The highest associations were found between BMI and the PACER laps and between WC and strength in obese children. As PF comprises various components, such as cardiovascular endurance, muscular strength, flexibility, and body composition, the impact of anthropometric and body composition measures may be more pronounced in some components and less in others. For example, BMI may have a more substantial influence on cardiovascular endurance (r = −0.073) compared to muscular strength (r = −0.56), as was found in our study. Studies also investigating similar associations confirmed that increased body mass contributes to decreases in aerobic fitness (Ding et al., 2020; Muller et al., 2022). As found in our study, reduced lower extremity muscle strength in OW and OB children may have contributed to their poor CRF, since obese children lack the essential muscle endurance for continuous running. In this regard, Bonney et al. (2018) reported that a loss of muscular strength and difficulty exercising can be reasons for decreasing CRF. The observed poor performance in the OB may also represent reduced activation of motor units (Wearing et al., 2006).

Low and very low BMIs were also associated with higher performance in other studies (Haywood et al., 2021; Verbecque-Alva et al., 2022; Xu et al., 2020). However, associations between BMI and aerobic capacity were insignificant in our UW and NW groups, although the strength of UW children was positively associated with BMI, WC and FFM, and in normal-weight children, BMI showed positive associations. Fat-free mass (FFM), which includes muscle, bone, water, and organ weight, is generally beneficial for strength and power.

Differences in strength between the OB group and the UW and normal weight groups can possibly be caused by structural changes to the body and an increase in the size of many body parts. This, in turn, increases the complexity and difficulty of non-stationary motions like jumping, sprinting, and lifting one’s body weight from a standing position (D’Hondt et al., 2012). Furthermore, a higher muscle-to-weight ratio in some UW children may improve strength-to-weight efficiency.

The UW group also completed noticeably more PACER laps, revealing significantly higher predicted $\dot{V}$ O_2_max (*p* < .001), speed and agility scores in the group compared not only to OW and obese OB children but also to normal-weight peers. Their lower body mass may result in more efficient movement, reducing physical strain and enabling faster and more agile performance. Energy conservation is also important because carrying less mass requires less energy, which is beneficial for endurance activities. According to the notion of developmental plasticity, OW and/or OB in children and adolescents can interfere with motor performance and affect postural control, impairing motor coordination (Barros et al., 2022). An explanation is that a bigger body is more difficult to move and manage during functional movements, such as in agility tests, standing long jumps and running. It requires more force, an appropriate stride length, foot stride, and balance, and the ability to change direction quickly (Barnett et al., 2016; Barros et al., 2022; Kohl et al., 2013). Psychological factors such as increased motivation or a desire to prove oneself may also have improved the UW group’s performance. Smith et al. (2020) found that OW schoolchildren with a mean age of 9.5 years performed significantly worse in lower body strength and cardiorespiratory tests than their UW and typical peers in a study to determine PF status and its association with BC, growth, and selected socio-demographics.

Less significant, although still clear, associations were also found between specific MS (balance and catching) and the various BC groups. The normal- and UW groups showed significant small to moderate positive correlations between BMI, WC, and FFM, and these skills, compared to the OB group, where negative and high associations were found between BMI, WC, and FFM and balance. These findings agree with the findings of Awad and Aneis (2022), who studied relationships between BMI and MS (balance, dropping and catching a ball) using the BOT-2 short-form, revealing higher skills scores in normal and UW children compared to OW or OB Egyptian children, aged 6.73 ± 0.75 years. Surprisingly, in our study, the OB group was the only group where the kicking accuracy was positively associated with WC and FFM. A possible explanation can be that having a higher BMI provides greater stability and balance, especially during the final stepping phase before kicking occurs. The extra weight may have led to a more grounded posture, allowing OW children to kick more accurately than UW and normal-weight children.

Our results also indicated no differences between underweight and children of normal weight in most of the test items. Instead, UW children revealed better scores in most of the HRPF, MRPF, and MS tests. These findings differ from a study on Egyptian children that reports inferior fitness and motor skills in UW children (Abdelkarim et al., 2020) and from Monyeki et al. (2005) on South African children, but concur with findings on children in Spain (Vandoni et al., 2021), where UW was not associated with fitness in children (Gulías-González et al., 2014). A possible explanation for the similar fitness of UW and NW children in our study is that the UW children included in this study rather have thin body statures and do not reflect a group of undernourished children, which may mask the true difference between UW and NW. Furthermore, impaired fitness or motor skill competence may only occur in severe cases. Thin but well-fed children may also present with low BMI for their age, but their body composition may be entirely different. In our study, the BMI of the UW group (15.13) was very similar to that of the normal weight group (15.60), as well as their WC. Their fat percentage (13.84 vs. 23.99) and fat (kg) (3.46 vs. 5.64) were significantly lower than in the NW group, although we assume that they did not represent severe forms of underweight. Their lean mass, including muscle mass, was also higher than in NW children, suggesting healthier metabolic health, thus contributing to better performance. In future research, thin but well-nourished children should be distinguished from undernourished children by not only focusing on an anthropometric BMI proxy for UW but also by combining other criterion methods for body composition. It is recommended that it should also be determined whether the severity of undernourishment and/or the age of the children is decisive in the development of also reduced physical fitness and motor skills difficulties. Factors such as type and severity of malnutrition may have masked the true extent of these differences, contributing to this variability. Literature that reports on fitness and motor skills in UW children is scarce. As such, we could not explore these findings comprehensively.

Overall, the results showed that a healthier weight, as found in UW and normal-weight children, positively influences fitness and motor skills more than OW and OB on the MS of children aged five to eight years. The results confirmed that physical and motor fitness and the quality and quantity of performing MS, such as balancing and catching, are already negatively influenced at a young age due to poor body composition characteristics such as an inflated BMI, WC, and FM. Therefore, OW and OB can negatively affect children’s involvement in sports and physical activities and their capacity to perform MS (Barros et al., 2022; Morano et al., 2020). This negative spiral can, in turn, impede PA levels (Barros et al., 2022; Morano et al., 2020). Children who lack the motivation to engage in physical activities, such as OB children, may have lower levels of actual and perceived physical competence, which can limit their potential to move to increase skilfulness and a sense of competence (Barros et al., 2022; Morano et al., 2020). It is therefore important to take a comprehensive approach involving multiple stakeholders, including schools, parents, healthcare providers, and community organisations, to create a supportive environment that encourages PA and healthy habits among young children, regardless of their weight or skill level.

### Strengths and Limitations

The strength of this study is that the associations of adiposity on fitness and motor skills in underweight and overweight children were studied by combining anthropometric and body composition measures using BIA, which is commonly used to assess body composition in clinical practice and scientific research. This approach advantages the findings when determining total body weight and ultimately FFM, FM and BF% or BC. In addition, the MS of five- to eight-year-old children were studied qualitatively and quantitatively, which is a further unique contribution of the study. Furthermore, it was conducted on children aged five to eight years old, which is an important age range for the development of positive activity behaviours and better nutritional choices that may have a long-term effect on the health and well-being of children. As limited studies are reported in this age group, particularly on South African children, this study strengthens the understanding of associations with body composition that require attention at this age, as revealed by the findings of this study. However, this study also had limitations that need to be acknowledged. The cross-sectional nature of the data and the fact that the findings are based on a sub-sample of the ExAMIN-Youth study limit the generalisability of the results to the larger population of the study and the whole of South Africa. In addition, cross-sectional data cannot be used to test causal relationships. Longitudinal studies with bigger sample sizes are recommended for a more in-depth understanding of causal relationships and to help identify critical periods in a child’s development when body fatness may significantly impact fitness and motor skills. The complexity of using VO_2_ max as a measure of aerobic fitness in young children is also acknowledged, as attention span, motivation, and developmental differences can affect the reliability of the results; therefore, alternative testing might have provided better results. However, we employed various efforts to reduce these influences. Participants were assisted in pacing themselves by having a research member run the 20-m shuttle run test with them until completion. They were also encouraged to keep going and run until exhaustion. Each participant was also consistently applauded for their effort from the side and at the 20-m turning points of the test for their effort by research members. More studies with large sample sizes are recommended in this area, as well as studies to identify interventions that can reduce OB through MS interventions.

## Conclusion

The findings confirmed that high body fatness was associated negatively with HRPF, MRPF and MS during early childhood, as large negative associations with OW and OB were found compared to moderate positive relationships between UW, NW, MRPF, HRPF, and MS. Clear associations of BMI, WC, and FFM were evident in MRPF, HRPF, and MS, although BF% did not affect these skills at this young age. These hampering effects of adiposity at the young age of five to eight years are concerning for various reasons. Children with low levels of gross motor competence due to adiposity tend to be less active later in their lives, with likely lower levels of cardiovascular fitness (CRF) and muscular fitness. Prevention strategies are therefore essential to combat and reduce childhood obesity, such as promoting healthy eating habits and physical activities at daycare centres, schools, and in households.

## References

[bibr1-00315125251348493] Abarca-Gómez L. Abdeen Z. A. Hamid Z. A. Abu-Rmeileh N. M. Acosta-Cazares B. Acuin C. Adams R. J. Aekplakorn W. Afsana K. Aguilar-Salinas C. A. Agyemang C. Ahmadvand A. Ahrens W. Ajlouni K. Akhtaeva N. Al-Hazzaa H. M. Al-Othman A. R. Al-Raddadi R. Al Buhairan F. Al Dhukair S. (2017). Worldwide trends in body-mass index, underweight, overweight, and obesity from 1975 to 2016: A pooled analysis of 2416 population-based measurement studies in 128·9 million children, adolescents, and adults. The Lancet, 390(10113), 2627–2642. 10.1016/s0140-6736(17)32129-3 PMC5735219 29029897

[bibr2-00315125251348493] Abdelkarim O. Ammar A. Trabelsi K. Cthourou H. Jekauc D. Irandoust K. Taheri M. Bös K. Woll A. Bragazzi N. L. Hoekelmann A. (2020). Prevalence of underweight and overweight and its association with physical fitness in Egyptian schoolchildren. International Journal of Environmental Research and Public Health, 17(1), 75. 10.3390/ijerph17010075 PMC6981920 31861878

[bibr3-00315125251348493] Allen L. Kelly B. B. (2019). Child development and early learning. Nih.gov. National Academies Press (US). https://www.ncbi.nlm.nih.gov/books/NBK310550/

[bibr4-00315125251348493] Artero E. G. España-Romero V. Ortega F. B. Jiménez-Pavón D. Ruiz J. R. Vicente-Rodríguez G. Bueno M. Marcos A. Gómez-Martínez S. Urzanqui A. González-Gross M. Moreno L. A. Gutiérrez A. Castillo M. J. (2010). Health-related fitness in adolescents: Underweight, and not only overweight, as an influencing factor. The AVENA study. Scandinavian Journal of Medicine & Science in Sports, 20(3), 418–427. 10.1111/j.1600-0838.2009.00959.x 19558383

[bibr5-00315125251348493] Awad A. S. Aneis Y. M. (2022). Correlation between body mass index and motor proficiency in Egyptian children: A cross-sectional study. Bulletin of Faculty of Physical Therapy, 27(1), 26–29. 10.1186/s43161-022-00087-7

[bibr6-00315125251348493] Baard M. (2014). Body mass index and associated physical activity levels in 7 - 10-year-old children in primary schools in Port Elizabeth. South African Journal of Sports Medicine, 26(4), 115–118. 10.7196/sajsm.551

[bibr7-00315125251348493] Bakaloudi D. R. Siargkas A. Poulia K. A. Dounousi E. Chourdakis M. (2020). The effect of exercise on nutritional status and body composition in hemodialysis: A systematic review. Nutrients, 12(10), 3071. 10.3390/nu12103071 33050111 PMC7601723

[bibr8-00315125251348493] Barnett L. M. Stodden D. Cohen K. E. Smith J. J. Lubans D. R. Lenoir M. Iivonen S. Miller A. D. Laukkanen A. Dudley D. Lander N. J. Brown H. Morgan P. J. (2016). Fundamental movement skills: An important focus. Journal of Teaching in Physical Education, 35(3), 219–225. 10.1123/jtpe.2014-0209

[bibr9-00315125251348493] Barros W. M. A. da Silva K. G. Silva R. K. P. Souza A. P. d. S. da Silva A. B. J. Silva M. R. M. Fernandes M. S. de Souza S. L. Souza V. d. O. N. (2022). Effects of overweight/obesity on motor performance in children: A systematic review. Frontiers in Endocrinology, 12, 759165–759214. 10.3389/fendo.2021.759165 35126307 PMC8812008

[bibr11-00315125251348493] Bonney E. Ferguson G. Smits-Engelsman B. (2018). Relationship between body mass index, cardiorespiratory and musculoskeletal fitness among South African adolescent girls. International Journal of Environmental Research and Public Health, 15(6), 1087. 10.3390/ijerph15061087 29843388 PMC6025162

[bibr12-00315125251348493] Bourne L. T. Lambert E. V. Steyn K. (2001). Where does the black population of South Africa stand on the nutrition transition? Public Health Nutrition, 5(1A), 157–162. 10.1079/PHN2001288 12027279

[bibr13-00315125251348493] Chen G. Chen J. Liu J. Hu Y. Liu Y. (2022). Relationship between body mass index and physical fitness of children and adolescents in Xinjiang, China: A cross-sectional study. BMC Public Health, 22(1), 1680. 10.1186/s12889-022-14089-6 36064657 PMC9442906

[bibr14-00315125251348493] Cheng J. East P. Blanco E. Sim E. K. Castillo M. Lozoff B. Gahagan S. (2016). Obesity leads to declines in motor skills across childhood. Child: Care, Health and Development, 42(3), 343–350. 10.1111/cch.12336 27059409 PMC4841726

[bibr15-00315125251348493] Children Count . (2019). Childrencount.uct.ac.za. https://childrencount.uct.ac.za/indicator.php?domain=4&indicator=96

[bibr16-00315125251348493] Choukem S. P. Tochie J. N. Sibetcheu A. T. Nansseu J. R. Hamilton-Shield J. P. (2020). Overweight/obesity and associated cardiovascular risk factors in sub-Saharan African children and adolescents: A scoping review. International Journal of Pediatric Endocrinology, 2020(6), 6–13. 10.1186/s13633-020-0076-7 32211050 PMC7092532

[bibr17-00315125251348493] Cohen J. (1988). Statistical power analysis for the behavioural sciences (2nd ed.). Erlbaum.

[bibr18-00315125251348493] Cole T. J. Flegal K. M. Nicholls D. Jackson A. A. (2007). Body mass index cut offs to define thinness in children and adolescents: International survey. BMJ, 335(7612), 194. 10.1136/bmj.39238.399444.55 17591624 PMC1934447

[bibr19-00315125251348493] de-Mateo-Silleras B. de-la-Cruz-Marcos S. Alonso-Izquierdo L. Camina-Martín M. A. Marugán-de-Miguelsanz J. M. Redondo-Del-Río M. P. (2019). Bioelectrical impedance vector analysis in obese and overweight children. PLoS One, 14(1), Article e0211148. 10.1371/journal.pone.0211148 30677103 PMC6345442

[bibr20-00315125251348493] de Onis M. Branca F. (2016). Childhood stunting: A global perspective. Maternal and Child Nutrition, 12(Suppl 1), 12–26. 10.1111/mcn.12231 27187907 PMC5084763

[bibr21-00315125251348493] de Onis M. Onyango A. W. Borghi E. Siyam A. Nishida C. Siekmann J. (2007). Development of a WHO growth reference for school-aged children and adolescents. Bulletin of the World Health Organization, 85(9), 660–667. 10.2471/blt.07.043497 18026621 PMC2636412

[bibr22-00315125251348493] Dereń K. Nyankovskyy S. Nyankovska O. Łuszczki E. Wyszyńska J. Sobolewski M. Mazur A. (2018). The prevalence of underweight, overweight and obesity in children and adolescents from Ukraine. Scientific Reports, 8(1), 1–7. 10.1038/s41598-018-21773-21774 29483604 PMC5826931

[bibr23-00315125251348493] de Waal E. Pienaar A. E. (2021). Influences of persistent overweight on perceptual-motor proficiency of primary schoolchildren: the North-West CHILD longitudinal study. BMC Pediatrics, 21(1), 245–310. 10.1186/s12887-021-02708-x 34016074 PMC8136082

[bibr24-00315125251348493] D’Hondt E. Deforche B. Gentier I. De Bourdeaudhuij I. Vaeyens R. Philippaerts R. Lenoir M. (2012). A longitudinal analysis of gross motor coordination in overweight and obese children versus normal-weight peers. International Journal of Obesity, 37(1), 61–67. 10.1038/ijo.2012.55 22508339

[bibr25-00315125251348493] Ding D. Mutrie N. Bauman A. Pratt M. Hallal P. R. C. Powell K. E. (2020). Physical activity guidelines 2020: Comprehensive and inclusive recommendations to activate populations. The Lancet, 396(10265), 1780–1782. 10.1016/s0140-6736(20)32229-7 33248019

[bibr26-00315125251348493] Drenowatz C. Hinterkörner F. Greier K. (2021). Physical fitness and motor competence in upper Austrian elementary schoolchildren: Study protocol and preliminary findings of a state-wide fitness testing program. Frontiers in Sports and Active Living, 3, 635478–635511. 10.3389/fspor.2021.635478 33693431 PMC7937611

[bibr27-00315125251348493] Fiori F. Bravo G. Parpinel M. Messina G. Malavolta R. Lazzer S. (2020). Relationship between body mass index and physical fitness in Italian prepubertal schoolchildren. PLoS One, 15(5), Article e0233362. 10.1371/journal.pone.0233362 32442183 PMC7244112

[bibr28-00315125251348493] Förster L. J. Vogel M. Stein R. Hilbert A. Breinker J. L. Böttcher M. Kiess W. Poulain T. (2023). Mental health in children and adolescents with overweight or obesity. BMC Public Health, 23(1), 135–211. 10.1186/s12889-023-15032-z 36658514 PMC9849834

[bibr29-00315125251348493] Gahche J. Fakhouri T. Carroll D. D. Burt V. L. Wang C.-Y. Fulton J. E. (2014). Cardiorespiratory fitness levels among U.S. Youth aged 12–15 years: United States, 1999-2004 and 2012. NCHS data brief, 153, 1–8. https://pubmed.ncbi.nlm.nih.gov/24871993/. 24871993

[bibr30-00315125251348493] Georgousakis M. (2019). Introducing the obesity evidence Hub. https://media.bupa.com.au/introducing-the-obesity-evidence-hub

[bibr31-00315125251348493] Gulías-González R. Martínez-Vizcaíno V. García-Prieto J. C. Díez-Fernández A. Olivas-Bravo A. Sánchez-López M. (2014). Excess of weight, but not underweight, is associated with poor physical fitness in children and adolescents from Castilla-La Mancha, Spain. European Journal of Pediatrics, 173(6), 727–735. 10.1007/s00431-013-2233-y 24326383

[bibr32-00315125251348493] Hall K. D. Ayuketah A. Brychta R. Cai H. Cassimatis T. Chen K. Y. Chung S. T. Costa E. Courville A. Darcey V. Fletcher L. A. Forde C. G. Gharib A. M. Guo J. Howard R. Joseph P. V. McGehee S. Ouwerkerk R. Raisinger K. Zhou M. (2019). Ultra-processed diets cause excess calorie intake and weight gain: An inpatient randomized controlled trial of ad libitum food intake. Cell Metabolism, 30(1), 67–77.e3. 10.1016/j.cmet.2019.05.008 31105044 PMC7946062

[bibr90-00315125251348493] Haywood X. Pienaar A. E. (2007). Long-Term Influences of Stunting Being Underweight, and Thinness on the Academic Performance of Primary School Girls: The NW-CHILD Study. Int. J. Environ. Res. Public Health, 18(8973). 10.3390/ijerph18178973 PMC8431266 34501563

[bibr33-00315125251348493] Huang Y.-C. Malina R. M. (2007). BMI and health-related physical fitness in Taiwanese youth 9-18 years. Medicine & Science in Sports & Exercise, 39(4), 701–708. 10.1249/mss.0b013e31802f0512 17414809

[bibr34-00315125251348493] Iivonen S. Sääkslahti A. k. (2013). Preschool children’s fundamental motor skills: A review of significant determinants. Early Child Development and Care, 184(7), 1107–1126. 10.1080/03004430.2013.837897

[bibr35-00315125251348493] Kahn M. E. Mohaddes K. Ng R. N. C. Pesaran M. H. Raissi M. Yang J. C. (2021). Long-term macroeconomic effects of climate change: A cross-country analysis. Energy Economics, 104(105624). 10.1016/j.eneco.2021.105624

[bibr36-00315125251348493] Kamruzzaman Md. Rahman S. A. Akter S. Shushmita H. Ali M. Y. Billah M. A. Kamal M. S. Elahi M. T. Paul D. K. (2021). The anthropometric assessment of body composition and nutritional status in children aged 2–15 years: A cross-sectional study from three districts in Bangladesh. PLoS One, 16(9), Article e0257055. 10.1371/journal.pone.0257055 34499671 PMC8428712

[bibr37-00315125251348493] Kohl L. F. Crutzen R. de Vries N. K. (2013). Online prevention aimed at lifestyle behaviors: A systematic review of reviews. Journal of Medical Internet Research, 15(7), Article e146. 10.2196/jmir.2665 23859884 PMC3714003

[bibr87-00315125251348493] Kruger R. Kruger H. S. Monyeki M. A. Pienaar A. E. Botha-Le Roux S. Gafane-Matemane L. F. Wayne Smith W. Catharina Martha Cornelia Mels C. M. C. Lammertyn L. Susanna Brits Johanna J.S. Hanssen H. (2021). A demographic approach to assess elevated blood pressure and obesity in prepubescent children: The ExAMIN Youth South Africa study. Journal of Hypertension, 39(11), 2190–2199. 10.1097/HJH.0000000000002917 34620809

[bibr38-00315125251348493] Kruger R. Monyeki M. A. Schutte A. E. Smith W. Mels C. M. C. Kruger H. S. Pienaar A. E. Gafane-Matemane L. F. Breet Y. Lammertyn L. Mokwatsi G. G. Kruger A. Deacon E. Hanssen H. (2020). The exercise, arterial modulation and nutrition in youth South Africa study (ExAMIN youth SA). Frontiers in Pediatrics, 8(212), 212. 10.3389/fped.2020.00212 32411640 PMC7201091

[bibr39-00315125251348493] Lima R. A. Bugge A. Ersbøll A. K. Stodden D. F. Andersen L. B. (2019). The longitudinal relationship between motor competence and measures of fatness and fitness from childhood into adolescence. Jornal de Pediatria, 95(4), 482–488. 10.1016/j.jped.2018.02.010 29782811

[bibr40-00315125251348493] Lima R. A. Soares F. C. Queiroz D. R. Aguilar J. A. Bezerra J. Barros M. V. G. (2021). The importance of body weight status on motor competence development: From preschool to middle childhood. Scandinavian Journal of Medicine & Science in Sports, 31(Suppl 1), 15–22. 10.1111/sms.13787 32735359 PMC8252800

[bibr41-00315125251348493] Livonen S. Sääkslahti A. K. (2013). Preschool children’s fundamental motor skills: A review of significant determinants. Early Child Development and Care, 184(7), 1107–1126. 10.1080/03004430.2013.837897

[bibr42-00315125251348493] Llewellyn A. Simmonds M. Owen C. G. Woolacott N. (2015). Childhood obesity as a predictor of morbidity in adulthood: A systematic review and meta-analysis. Obesity Reviews, 17(1), 56–67. 10.1111/obr.12316 26440472

[bibr43-00315125251348493] Luke A. Durazo-Arvizu R. Rotimi C. Prewitt T. E. Forrester T. Wilks R. Ogunbiyi O. J. Schoeller D. A. McGee D. Cooper R. S. (1997). Relation between body mass index and body fat in black population samples from Nigeria, Jamaica, and the United States. American Journal of Epidemiology, 145(7), 620–628. 10.1093/oxfordjournals.aje.a009159 9098179

[bibr44-00315125251348493] McCarthy H. D. Cole T. J. Fry T. Jebb S. A. Prentice A. M. (2006). Body fat reference curves for children. International Journal of Obesity, 30(4), 598–602. 10.1038/sj.ijo.0803232 16570089

[bibr45-00315125251348493] Meriem C. Khaoula M. Ghizlane C. Asmaa M. A. Ahmed A. O. T. (2020). Early childhood development (0 - 6 Years old) from healthy to pathologic: A review of the literature. Open Journal of Medical Psychology, 09(03), 100–122. 10.4236/ojmp.2020.93009

[bibr46-00315125251348493] Modjadji P. Madiba S. (2022). The multidimension of malnutrition among schoolchildren in a rural area, South Africa: A mixed methods approach. Nutrients, 14(23), 5015. 10.3390/nu14235015 36501045 PMC9741400

[bibr47-00315125251348493] Moeng-Mahlangu L. T. Monyeki M. A. Reilly J. J. Mchiza Z. J. Moleah T. Loechl C. U. Kruger H. S. (2020). Level of agreement between objectively determined body composition and perceived body image in 6- to 8-year-old South African children: The Body Composition–Isotope Technique study. PLoS One, 15(8), Article e0237399. 10.1371/journal.pone.0237399 32777810 PMC7417193

[bibr48-00315125251348493] Monteiro C. A. Conde W. L. Popkin B. M. (2002). Is obesity replacing or adding to undernutrition? Evidence from different social classes in Brazil. Public Health Nutrition, 5(1A), 105–112. 10.1079/PHN2001281 12027272

[bibr49-00315125251348493] Monyeki M. A. Awotidebe A. Strydom G. L. de Ridder J. H. Mamabolo R. L. Kemper H. C. (2015). The challenges of underweight and overweight in South African children: Are we winning or losing the battle? A systematic review. International Journal of Environmental Research and Public Health, 12(2), 1156–1173. 10.3390/ijerph120201156 25648175 PMC4344660

[bibr50-00315125251348493] Monyeki M. A. Koppes L. L. J. Kemper H. C. G. Monyeki K. D. Toriola A. L. Pienaar A. E. Twisk J. W. R. (2005). Body composition and physical fitness of undernourished South African rural primary schoolchildren. European Journal of Clinical Nutrition, 59(7), 877–883. 10.1038/sj.ejcn.1602153 15915157

[bibr51-00315125251348493] Monyeki M. A. Neetens R. Moss S. J. Twisk J. (2012). The relationship between body composition and physical fitness in 14 year old adolescents residing within the Tlokwe local municipality, South Africa: The PAHL study. BMC Public Health, 12(1), 374. 10.1186/1471-2458-12-374 22626033 PMC3461487

[bibr52-00315125251348493] Morano M. Robazza C. Bortoli L. Rutigliano I. Ruiz M. C. Campanozzi A. (2020). Physical activity and physical competence in overweight and obese children: An intervention study. International Journal of Environmental Research and Public Health, 17(17), 6370. 10.3390/ijerph17176370 32883044 PMC7504542

[bibr53-00315125251348493] Müller A. Nagy Z. Kovács S. Szőke S. Bendíková E. Ráthonyi G. Ráthonyi-Ódor K. Szabados G. Gabnai Z. Bába É. B. (2022). Correlations between physical fitness and body composition among boys aged 14–18 conclusions of a case study to reverse the worsening secular trend in fitness among urban youth due to sedentary lifestyles. International Journal of Environmental Research and Public Health, 19(14), 8765. 10.3390/ijerph19148765 35886622 PMC9323754

[bibr54-00315125251348493] Müllerova H. Maselli D. J. Locantore N. Vestbo J. Hurst J. R. Wedzicha J. A. Bakke P. Agusti A. Anzueto A. (2015). Hospitalized exacerbations of COPD: Risk factors and outcomes in the ECLIPSE cohort. Chest (American College of Chest Physicians), 147(4), 999–1007. 10.1378/chest.14-0655 25356881

[bibr55-00315125251348493] National Department of Health (NdoH) . (2018). National Health Survey. Demographic and health survey. 2016. Key findings Statistics South Africa South African Medical Research Council and ICF. Statistics South Africa (stats SA), 1–19.

[bibr56-00315125251348493] Nobre J. N. P. Morais RLDS Fernandes A. C. Viegas Â. A. Figueiredo P. H. S. Costa H. S. Camargos A. C. R. Dias-Peixoto M. F. Mendonça V. A. Lacerda A. C. R. (2022). Is body fat mass associated with worse gross motor skills in preschoolers? An exploratory study. PLoS One, 17(3), Article e0264182. 10.1371/journal.pone.0264182 35263353 PMC8906635

[bibr57-00315125251348493] Otitoola O. Oldewage-Theron W. Egal A. (2020). Prevalence of overweight and obesity among selected school children and adolescents in Cofimvaba, South Africa. South African Journal of Clinical Nutrition, 34(3), 97–102. 10.1080/16070658.2020.1733305

[bibr58-00315125251348493] Pelletier D. L. Frongillo E. A. (2003). Changes in child survival are strongly associated with changes in malnutrition in developing countries. The Journal of Nutrition, 133(1), 107–119. 10.1093/jn/133.1.107 12514277

[bibr59-00315125251348493] Pienaar A. E. van Reenen I. Weber A. M. (2016). Sex differences in fundamental movement skills of a selected group of 6-year-old South African children. Early Child Development and Care, 186(12), 1994–2008. 10.1080/03004430.2016.1146263

[bibr88-00315125251348493] Plowman S. Meredith M . (2021). FITNESSGRAM ® /ACTIVITYGRAM ® Reference Guide (4 th Edition) [Internet]. Available from: https://fitnessgram.net/wp-content/uploads/2023/01/FITNESSGRAMACTIVITYGRAMReferenceGuide4thEd043015.pdf. 10.1097/HJH.0000000000002917

[bibr60-00315125251348493] Prista A. Daca T. Tchonga F. Machava E. Macucule C. Ribeiro E. (2016). Results from the Mozambique 2016 report card on physical activity for children and adolescents. Journal of Physical Activity and Health, 13(11 Suppl 2), S213–S217. 10.1123/jpah.2016-0526 27848722

[bibr61-00315125251348493] Raghuveer G. Hartz J. Lubans D. R. Takken T. Wiltz J. L. Mietus-Snyder M. Perak A. M. Baker-Smith C. Pietris N. Edwards N. M. American Heart Association Young Hearts Athero, Hypertension and Obesity in the Young Committee of the Council on Lifelong Congenital Heart Disease and Heart Health in the Young . (2020). Cardiorespiratory fitness in youth: An important marker of health: A scientific statement from the American heart association. Circulation (New York, N. Y.), 142(7), e101–e118. 10.1161/cir.0000000000000866 PMC7524041 32686505

[bibr62-00315125251348493] Ratajczak J. Petriczko E. (2020). The predictors of obesity among urban girls and boys aged 8–10 years: A cross-sectional study in North-Western Poland. International Journal of Environmental Research and Public Health, 17(18), 6611. 10.3390/ijerph17186611 32932779 PMC7559587

[bibr63-00315125251348493] Robinson L. E. Stodden D. F. Barnett L. M. Lopes V. P. Logan S. W. Rodrigues L. P. D’Hondt E. (2015). Motor competence and its effect on positive developmental trajectories of health. The Journal of Sports Medicine, 45(9), 1273–1284. 10.1007/s40279-015-0351-6 26201678

[bibr91-00315125251348493] Rudd J. Butson M. L. Barnett L. Farrow D. Berry J. Borkoles E. Polman R. (2016). A holistic measurement model of movement competency in children. Journal of Sports Sciences, 34(5), 477–485. 10.1080/02640414.2015.1061202 26119031

[bibr64-00315125251348493] Ruiz J. R. Castro-Piñero J. Artero E. G. Ortega F. B. Sjöström M. Suni J. Castillo M. J. (2009). Predictive validity of health-related fitness in youth: A systematic review. British Journal of Sports Medicine, 43(12), 909–923. 10.1136/bjsm.2008.056499 19158130

[bibr65-00315125251348493] Said M. A. Alhumaid M. M. Atta I. I. Al-Sababha K. M. Abdelrahman M. A. Alibrahim M. S. (2022). Lower fitness levels, higher fat-to-lean mass ratios, and lower cardiorespiratory endurance are more likely to affect the body mass index of Saudi children and adolescents. Frontiers in Public Health, 10(984469). 10.3389/fpubh.2022.984469 PMC9582435 36276343

[bibr66-00315125251348493] Saleemi M. A. Ashraf R. N. Mellander L. Zaman S. (2001). Determinants of stunting at 6, 12, 24 and 60 months and postnatal linear growth in Pakistani children. Acta Paediatrica, 90(11), 1304–1308. 10.1080/080352501317130371 11808904

[bibr67-00315125251348493] Schoeller D. A. Santen E. Peterson D. W. Dietz W. Jaspan J. Klein P. D. (1980). Total body water measurement in humans with 18O and 2H labeled water. American Journal of Clinical Nutrition, 33(12), 2286–2293. 10.1093/ajcn/33.12.2686 6776801

[bibr68-00315125251348493] Shang X. Liu A. Li Y. Hu X. Du L. Ma J. Xu G. Li Y. Guo H. Ma G. (2010). The association of weight status with physical fitness among Chinese children. International Journal of Pediatrics, 2010, 515414–515416. 10.1155/2010/515414 20652083 PMC2905728

[bibr69-00315125251348493] Smartspeed Fusion Sports, Summer Park, Brisbane, Australia . https://www.google.com/url?sa=t&rct=j&q=&esrc=s&source=web&cd=&ved=2ahUKEwjKyeGA_pr5AhVEoVwKHXwECmUQFnoECBEQAQ&url=https%3A%2F%2Fmanuals.plus%2Fm%2F87d075813d882539dd8d36e4bb4fcc048c4e48e3323920613f216791df0b7dc9_optim.pdf&usg=AOvVaw2WQ5MUNr7yfFobM_Y6boVt (accessed on 20 January 2020).

[bibr70-00315125251348493] Smith J. D. Fu E. Kobayashi M. A. (2020). Prevention and management of childhood obesity and its psychological and health comorbidities. Annual Review of Clinical Psychology, 16(1), 351–378. 10.1146/annurev-clinpsy-100219-060201 PMC7259820 32097572

[bibr89-00315125251348493] StatsSA . Republic of South Africa. Demographics (Statistics South Africa). Pretoria: National Department of Statistics. (2022). Available at https://www.statssa.gov.za/ (accessed November 2022). [Google Scholar].

[bibr71-00315125251348493] Stewart A. D. Benson P. J. Olds T. Marfell-Jones M. MacSween A. Nevill A. M. (2010). Self-selection of athletes into sports via skeletal ratios (pp. 307–322). Nova Science Publishers.

[bibr72-00315125251348493] Stodden D. F. Goodway J. D. Langendorfer S. J. Roberton M. A. Rudisill M. E. Garcia C. Garcia L. E. (2008). A developmental perspective on the role of motor skill competence in physical activity: An emergent relationship. Quest, 60(2), 290–306. 10.1080/00336297.2008.10483582

[bibr73-00315125251348493] Tomkinson G. R. Lang J. J. Blanchard J. Léger L. A. Tremblay M. S. (2019). The 20-m shuttle run: Assessment and Interpretation of data in relation to youth aerobic fitness and health. Pediatric Exercise Science, 31(2), 152–163. 10.1123/pes.2018-0179 30885058

[bibr74-00315125251348493] Ulrich D. A. (2000). Test of gross motor development 2: Examiner’s manual (2nd ed.). PRO-ED.

[bibr75-00315125251348493] Utesch T. Dreiskämper D. Naul R. Geukes K. (2018). Understanding physical (in-) activity, overweight, and obesity in childhood: Effects of congruence between physical self-concept and motor competence. Scientific Reports, 8(1), 5908. 10.1038/s41598-018-24139-y 29651046 PMC5897370

[bibr76-00315125251348493] Vandoni M. Calcaterra V. Carnevale Pellino V. De Silvestri A. Marin L. Zuccotti G. V. Tranfaglia V. Giuriato M. Codella R. Lovecchio N. (2021). Fitness and Fatness’ in children and adolescents: An Italian cross-sectional study. Children, 8(9), 762. 10.3390/children8090762 34572192 PMC8470229

[bibr77-00315125251348493] Velázquez-Alva M. C. Irigoyen-Camacho M. E. Zepeda-Zepeda M. A. Rangel-Castillo I. Arrieta-Cruz I. Mendoza-Garcés L. Castaño-Seiquer A. Flores-Fraile J. Gutiérrez-Juárez R. (2022). Comparison of body fat percentage assessments by bioelectrical impedance analysis, anthropometrical prediction equations, and dual-energy X-ray absorptiometry in older women. Frontiers in Nutrition, 9(978971). 10.3389/fnut.2022.978971 PMC9812576 36618693

[bibr78-00315125251348493] Wang Z. Deurenberg P. Wang W. Pietrobelli A. Baumgartner R. N. Heymsfield S. B. (1999). Hydration of fat free body mass: Review and critique of a classic body-composition constant. American Journal of Clinical Nutrition, 69(5), 833–841. 10.1093/ajcn/69.5.833 10232621

[bibr79-00315125251348493] Wearing S. C. Hennig E. M. Byrne N. M. Steele J. R. Hills A. P. (2006). The impact of childhood obesity on musculoskeletal form. Obesity Reviews, 7(2), 209–218. 10.1111/j.1467-789X.2006.00216.x 16629876

[bibr80-00315125251348493] Wilhelmsen T. Røysamb E. Lekhal R. Brandlistuen R. E. Alexandersen N. Wang M. V. (2023). Children’s mental health: The role of multiple risks and child care quality. Journal of Applied Developmental Psychology, 86(101546). 10.1016/j.appdev.2023.101546

[bibr81-00315125251348493] Williams D. P. Going S. B. Lohman T. G. Harsha D. W. Srinivasan S. R. Webber L. S. Berenson G. S. (1992). Body fatness and risk for elevated blood pressure, total cholesterol, and serum lipoprotein ratios in children and adolescents. American Journal of Public Health, 82(3), 358–363. 10.2105/ajph.82.3.358 1536350 PMC1694353

[bibr82-00315125251348493] Wolnicka K. Jarosz M. Jaczewska-Schuetz J. Taraszewska A. (2016). Differences in the prevalence of overweight, obesity and underweight among children from primary schools in rural and urban areas. Annals of Agricultural and Environmental Medicine, 23(2), 341–344. 10.5604/12321966.1203902 27294644

[bibr83-00315125251348493] World Health Organization . (2023). Malnutrition. Who.int. World Health Organization: WHO. https://www.who.int/news-room/fact-sheets/detail/malnutrition

[bibr84-00315125251348493] World Health Organization . (2021). Malnutrition. https://www.who.int/news-room/fact-sheets/detail/malnutrition

[bibr85-00315125251348493] Wyszyńska J. Ring-Dimitriou S. Thivel D. Weghuber D. Hadjipanayis A. Grossman Z. Ross-Russell R. Dereń K. Mazur A. (2020). Physical activity in the prevention of childhood obesity: The position of the European childhood obesity group and the European academy of pediatrics. Frontiers in Pediatrics, 8, 535705–535708. 10.3389/fped.2020.535705 33224905 PMC7674497

[bibr86-00315125251348493] Xu Y. Mei M. Wang H. Yan Q. He G. (2020). Association between weight status and physical fitness in Chinese mainland children and adolescents: A cross-sectional study. International Journal of Environmental Research and Public Health, 17(7), 2468. 10.3390/ijerph17072468 32260379 PMC7177678

